# Health system disruption and oncologic consequences: a retrospective observational study of South Korea’s 2024 medical walkout

**DOI:** 10.12701/jyms.2026.43.4

**Published:** 2025-12-18

**Authors:** Seung Ho Song, Chang Hyun Kim, Soo Yeun Park

**Affiliations:** 1 Colorectal Cancer Center, Kyungpook National University Chilgok Hospital, Daegu, Korea; 2 Department of Surgery, School of Medicine, Kyungpook National University, Daegu, Korea; 3 Department of Surgery, Kyungpook National University Hospital, Daegu, Korea; 4 Department of Surgery, Chonnam National University Hwasun Hospital and Medical School, Hwasun, Korea

**Keywords:** Colorectal neoplasms, Delivery of health care, Health personnel, Health policy, Time-to-treatment

## Abstract

**Background:**

In February 2024, a sudden government policy to drastically increase medical school admissions triggered the mass resignations of medical trainees across South Korea, severely disrupting hospital operations. This study aimed to evaluate the impact of the resulting healthcare workforce disruptions on short-term clinical outcomes and the timing of colorectal cancer surgeries.

**Methods:**

This retrospective comparative study analyzed patients with colorectal cancer treated at two national university hospitals in Daegu and Gwangju, South Korea. Patients who first visited the colorectal surgery department between March and August of 2023 and 2024 were included. Data from 2020 to 2022 were used for extended comparisons. The primary outcome was the interval from initial outpatient visit to surgery. Secondary outcomes included treatment modality distribution, tumor staging, and postoperative complications.

**Results:**

A total of 895 patients in 2023 and 853 in 2024 were included. In 2024, only 39.5% of patients (337/853) underwent upfront surgery compared to 63.5% (569/895) in 2023. The median time to surgery increased from 30 days (interquartile range [IQR], 22–44 days) to 52 days (IQR, 30–72 days) (*p*=0.001). Clinical T3–4 tumors increased from 49.9% to 59.3% (*p*=0.018), lymph node-positive cases increased from 25.9% to 51.3% (*p*=0.001), and postoperative complication rates increased from 12.0% to 28.2% (*p*=0.001).

**Conclusion:**

The abrupt healthcare workforce crisis in early 2024 significantly delayed colorectal cancer surgeries and was associated with worse short-term oncologic outcomes. These findings highlight the critical importance of maintaining a stable healthcare workforce to protect access to timely cancer care.

## Introduction

Medical strikes have occurred in various countries due to disputes over working conditions, wages, and healthcare policies, including in the United Kingdom, where doctors protested contract changes affecting pay and working hours, and in Kenya, where strikes were driven by unmet agreements on remuneration, staffing, and hospital funding [1,2]. In South Korea, a major strike occurred in 2020 in response to government policies that proposed increasing medical school admissions by 400 students annually for 10 years, establishing a public medical school, integrating traditional Korean medicine into the national health insurance system, and legalizing telemedicine [3]. The strike lasted for approximately 17 days, during which young doctors, including residents, interns, and fellows, walked out of their training hospitals and medical students boycotted their classes.

In 2024, the South Korean government suddenly announced a policy to increase medical school admissions by 2,000 students starting the following year, representing a 67% increase from the existing annual enrollment of 3,058 students [4,5]. Unlike in 2020, when doctors staged a strike, most young doctors in 2024 chose to resign from their positions. More than 90% of resident physicians resigned and over 70% of medical students took a leave of absence, resulting in a severe shortage of medical personnel in teaching hospitals [6]. The policy, which was formulated without reflecting the voices of frontline medical professionals, sparked widespread resistance among young doctors, exacerbating frustration and uncertainty regarding the future. Rather than engaging in collective action, they opted to terminate their training contracts and leave their hospitals by February 20, 2024.

Tertiary hospitals, which account for most teaching hospitals in South Korea, experienced significant disruptions, one of which was a prolonged waiting time for surgeries. The sudden shortage of both surgeons and anesthesiologists reduced operating room capacity to 30% to 50% of normal levels. This study investigated the impact of such medical policy conflicts on the timing of colorectal cancer surgeries by reviewing data from two national university hospitals in South Korea.

## Methods

**Ethics statement:** This study (Project No. KNUCRC-25CR04) was conducted in accordance with the principles outlined in the Declaration of Helsinki and was approved by the Institutional Review Boards (IRBs) of Kyungpook National University Chilgok Hospital (KNUCH IRB 2025-03-010) and Chonnam National University Hwasun Hospital (CNUHH-2025-074). Informed consent was waived by the IRB because the data were deidentified.

### 1. Study design and participants

This study retrospectively analyzed patients diagnosed with colorectal cancer who visited the colorectal surgery departments of two national university hospitals: Kyungpook National University Hospital, Kyungpook National University Chilgok Hospital, and Chonnam National University Hwasun Hospital. The inclusion criteria were as follows: (1) newly diagnosed colorectal cancer and (2) first presentation to the colorectal surgery outpatient clinic of one of the participating tertiary hospitals. The exclusion criteria were as follows: (1) initial presentation to other clinical departments, (2) first presentation through the emergency department, (3) recurrent colorectal cancer, and (4) presence of another active primary malignancy. The patients were divided into two groups based on two distinct periods: March 2023 to August 2023 and March 2024 to August 2024. These intervals were selected because the workforce shortage was most severe between March 2024 and August 2024. After August 2024, each hospital adopted mitigation strategies that partially restored its surgical capacity, which could confound assessment of the direct impact of the disruption. Patients who first visited departments other than colorectal surgery were excluded from the analysis. Comparisons between the two groups focused on baseline characteristics, clinical tumor stages, initial treatment decisions, and the time from the first outpatient visit to treatment initiation or surgery. Among patients for whom upfront surgery was decided as the initial treatment, further analysis included additional variables, such as length of hospital stay, postoperative complications within 30 days, and pathological tumor stage. In this study, one operating room unit was defined as a single surgical session, typically lasting approximately 4 hours, either in the morning or afternoon; a full day of operating room use corresponded to two units. From March 2024, owing to workforce shortages, the number of weekly operating room units available for colorectal cancer surgeries was significantly reduced from 18 to 10 units per week at one hospital and from 12 to six units at the other.

### 2. Data collection and variables

The patient data included sex, age, American Society of Anesthesiologists physical status classification, tumor location (i.e., colon, rectum, or synchronous), initial serum carcinoembryonic antigen levels, clinical stage (as determined by computed tomography or magnetic resonance imaging), and initial treatment decisions made by the colorectal surgeon during the outpatient visit. Treatment decisions were categorized as follows: upfront surgery, chemotherapy, chemoradiotherapy, or other approaches including endoscopic resection, observation, referral to another hospital (either by patient choice or physician recommendation), loss to follow-up, and management of emergency situations related to colorectal cancer. Additionally, we analyzed the duration from the first outpatient visit to surgery, length of postoperative hospital stay, postoperative complications within 30 days (graded according to the Clavien–Dindo classification) [7], and pathological findings from surgical specimens.

### 3. Extended analysis

The study extended its analysis to include data from five distinct periods (March to August 2020, 2021, 2022, 2023, and 2024) using the same evaluation methods across all time points.

### 4. Hypothesis and primary outcome

In this study, we hypothesized that recent medical policies prolong waiting times for colorectal cancer surgeries. Accordingly, the primary outcome was defined as the duration between the initial outpatient visit to the colorectal surgery department and the date of surgery.

### 5. Secondary outcomes

Secondary outcomes included the distribution of initial treatment modalities; interval from the initial outpatient visit to the initiation of treatment; clinical and pathological tumor stages; pathological tumor characteristics; and postoperative outcomes, including complication rates.

### 6. Statistical analysis

Descriptive statistics were used to summarize patient demographics and clinical characteristics. Continuous variables were assessed for normality using the Shapiro–Wilk test. Normally distributed variables are presented as means with standard deviations, whereas non-normally distributed variables are presented as medians with interquartile ranges (IQRs). Categorical variables are reported as frequencies and percentages and compared using the chi-square or Fisher exact test, as appropriate. Comparisons of continuous variables between groups were conducted using the independent t-tests for normally distributed data and the Mann–Whitney U tests for non-normally distributed data. The primary endpoint, the duration from the initial outpatient visit to surgery, is reported as the median (IQR) and compared between years using the Mann–Whitney U test. To identify independent risk factors associated with postoperative complications, univariate and multivariable logistic regression analyses were performed, and the results are expressed as hazard ratios (HRs) with 95% confidence intervals (CIs). Variables with *p*<0.10 in the univariate analysis were included in the multivariable model. Data distributions are shown using box plots for time intervals and bar charts for treatment modalities. Extended analyses incorporating data from March to August 2020, 2021, 2022, 2023, and 2024 were conducted using the same statistical approach to examine temporal trends. Extended analyses for 2020 to 2024 were descriptive in nature, and the overall year-to-year differences were assessed using the same group comparison tests described above. All statistical analyses were performed using R statistical software (ver. 4.4.1; R Foundation for Statistical Computing, Vienna, Austria; https://www.r-project.org). A two-sided *p*-value<0.05 was considered statistically significant.

## Results

Between March 2023 and August 2023, among the 895 patients with colorectal cancer who first visited the colorectal surgery departments, 569 (63.5%) underwent upfront surgery, 111 (12.4%) received chemotherapy, and 155 (17.3%) received concurrent chemoradiotherapy (Table 1). In 2024, the proportion of patients undergoing upfront surgery significantly decreased to 337 of the 853 patients (39.5%), while 76 (8.9%) received chemotherapy and 157 (18.4%) received chemoradiotherapy (*p*=0.001). Additionally, the proportion of patients receiving other forms of treatment, including observation, endoscopic resection, or referral to another hospital, increased from 6.7% in 2023 to 33.2% in 2024. The median times from the first outpatient visit to the initiation of surgery, chemotherapy, or chemoradiotherapy at the same hospital were 30.0 days (IQR, 21–50 days) in 2023 and 36.0 days (IQR, 22–68 days) in 2024 (*p*=0.001). Extended comparisons showed that the median time to treatment was 19.5 days in 2020, 23.0 days in 2021, and 29.0 days in 2022 (Supplementary Table 1).

**Fig. 1.**
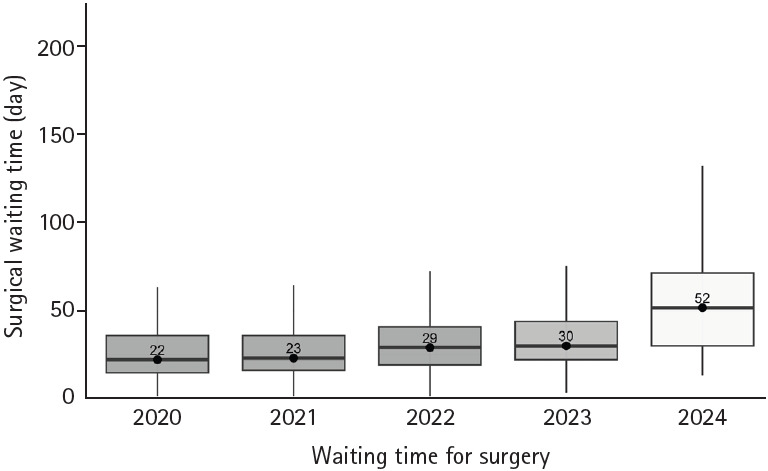
Median surgical waiting time for colorectal cancer by year.

Among patients who underwent surgery, the proportion of advanced tumors (clinical stage T3–4) increased from 49.9% in 2023 to 59.3% in 2024 (*p*=0.018), and the proportion of lymph node-positive cases increased from 25.9% to 51.3% (*p*=0.001). The median time from the first outpatient visit to surgery was 30 days (IQR, 22–44 days) in 2023 and 52 days (IQR, 30–72 days) in 2024 (*p*=0.001) (Table 2). Extended analysis showed that the median time to surgery in 2020, 2021, and 2022 was 22, 23, and 29 days, respectively (Fig. 1, Supplementary Table 2). The overall rate of postoperative complications increased from 12.0% in 2023 to 28.2% in 2024 (*p*=0.001). Multivariable analysis identified male sex (HR, 1.96; 95% CI, 1.51–2.57; *p*<0.001), rectal cancer (HR, 1.80; 95% CI, 1.38–2.34; *p*<0.001), and surgery performed in 2024 (HR, 3.50; 95% CI, 2.64–4.64; *p*<0.001) as independent predictors of postoperative complications (Table 3).

## Discussion

This study evaluated the impact of the large-scale healthcare workforce disruption, caused by a sudden national medical policy change, on colorectal cancer care at two national university hospitals in South Korea. The findings demonstrated significant shifts in clinical practice during the 2024 crisis, including a notable decline in upfront surgeries, increased use of alternative initial treatments, prolonged time to surgery, and higher rates of postoperative complications. Amid this disruption, a national media report highlighted a marked decrease in cancer surgeries following the mass resignation of medical trainees in early 2024, raising public concern about delays in cancer care across the country [8]. While such reports reflect the scale of the crisis, to our knowledge, no prior study has empirically examined how these events altered real-world surgical timelines or treatment decision-making. By quantifying delays in treatment initiation and characterizing changes in initial management strategies, this study offers important insights into the practical consequences of workforce instability on cancer care delivery.

The proportion of patients undergoing upfront surgery decreased significantly in 2024 compared with 2023, while the number of patients receiving alternative treatments, such as observation, endoscopic resection, or referral to another hospital, increased. This shift in treatment decision-making may be attributed to hospital resource constraints, particularly reduced operating room capacity following the mass resignations of resident physicians in both the surgical and anesthesiology departments. From March 2024, operating room availability for colorectal cancer surgeries was substantially reduced at both participating institutions; one hospital reduced its weekly allocation from 18 to 10 units and the other from 12 to six units. The reduction in operative capacity reflected not only shortages for colorectal surgery but also concurrent deficits in anesthesiology staffing. Because anesthesiology coverage determines operating room availability and permissible operating hours, limitations in both workforce groups interacted to amplify the overall disruption in the surgical workflow. In addition, both hospitals were required to start all surgeries at 8:00 a.m. and complete them by 4:00 p.m., as mandated by the anesthesiology departments, to manage the limited staffing. These combined limitations likely contributed to delays in surgical scheduling and the observed shifts in initial treatment strategies. During this period, outpatient decision-making was also influenced by resource limitations, introducing a potential selection bias. The marked increase in the “other” treatment category, particularly referrals, likely reflects the redirection of patients to external hospitals with available operative capacity.

Furthermore, the median time from the first outpatient visit to upfront surgery increased from 30 days in 2023 to 52 days in 2024, reflecting the impact of workforce shortages on surgical scheduling. Extended analysis revealed that waiting times in previous years (2020–2022) were considerably shorter, suggesting that the delays observed in 2024 were not part of a preexisting trend but rather a direct consequence of the recent policy-driven healthcare disruption. Although the median waiting time increased numerically between 2020 and 2023, this change was not statistically significant in contrast to the marked and statistically significant prolongation observed in 2024. This distinction further supports the notion that the delay in 2024 reflects an abrupt system-level disruption rather than a gradual secular trend.

Among the patients who underwent upfront surgery, the proportion with advanced clinical T-stage tumors and lymph node-positive disease was significantly higher in 2024 than in 2023. Additionally, pathological stage III–IV tumors were observed in 49.6% of the cases in 2024 compared with 38.1% in 2023, indicating a higher disease burden at the time of surgery. There are several possible explanations for these findings. Prolonged surgical delays in 2024 may have led to a shift in treatment patterns, wherein patients with early-stage colorectal cancer were more likely to be referred to secondary hospitals or specialized colorectal surgery centers capable of providing earlier surgical intervention. Additionally, the potential role of surgical delays in cancer progression cannot be ruled out, and further research using nationwide databases is warranted to confirm this association. Additional studies are required to assess the long-term oncological impact of such delays in colorectal cancer surgery.

The postoperative complication rate was significantly higher in 2024 than in 2023. Multivariable analysis identified male sex, rectal cancer, and surgery performed in 2024 as significant risk factors for postoperative complications. Because this was a retrospective study, the exact cause of the increased complication rate could not be definitively determined; however, several contributing factors may be considered. First, the higher proportion of patients with advanced-stage colorectal cancers undergoing surgery in 2024 may have contributed to the increased complication rate. Additionally, the mass resignation of junior doctors forced the remaining medical staff to shoulder more frequent on-call duties and increased workloads, potentially leading to higher fatigue and adversely affecting surgical outcomes. However, the proportion of severe complications (Clavien–Dindo grade III or higher) was significantly lower in 2024 (16.8%) than in 2023 (26.5%). Thus, although the overall complication rate increased, the severity profile did not worsen, as reflected by the lower proportion of Clavien–Dindo grade III–IV events in 2024. This may be partly related to changes in the case mix during the disruption and the reduced operative volume, which allowed the remaining surgical teams to focus more on each procedure, although the exact mechanism could not be determined. Therefore, further research is required to better understand the factors contributing to these postoperative complications. A study comparing postoperative complications after gastrectomy for gastric cancer during the same period (February–June) across 2021, 2022, 2023, and 2024 indicated no significant difference in postoperative complication rates [9]. The authors hypothesized that the reduced number of daily surgeries allowed for a greater focus on each case, which may have mitigated the impact of workforce shortages on complication rates. However, the effects of these disruptions on healthcare staffing may become more pronounced over time. Accumulating fatigue among the remaining medical staff, additional resignations, and limited support from other departments are likely to have a compounding effect. Since our study analyzed data through August, these cumulative effects may have contributed to the significant difference in complication rates. Additionally, while the median length of hospital stay was 7 days in both years, the corresponding IQRs indicated a relatively longer hospitalization period in 2024 (IQR in 2024, 6–9 days; IQR in 2023, 6–8 days). This indicates that the abrupt absence of junior doctors, including residents who played crucial roles in perioperative patient management, may have contributed to the prolonged postoperative care or monitoring period.

These findings highlight the critical role of healthcare workforce stability in ensuring timely and effective cancer treatment. While previous research has documented the impact of medical strikes on patient care, this study uniquely demonstrates how the large-scale resignation of resident physicians, rather than a traditional strike, can lead to significant surgical delays and potentially worsen disease status at the time of treatment. These results underscore the necessity for health policy decisions to incorporate the perspectives of frontline medical professionals to prevent unintended disruptions to essential healthcare services. Furthermore, this study illustrates the real-world consequences of implementing medical policies without consensus or adequate engagement with the medical community. Beyond the immediate and visible impact on surgical delays, there are growing concerns regarding the demoralization of young physicians and their reluctance to continue training in an increasingly uncertain and strained environment. This finding has profound implications for the future of South Korea’s healthcare system. The current generation of senior medical professionals working in university hospitals will inevitably retire in the coming years, and the next generation will need training to sustain the healthcare system. Cancer treatment, in particular, requires a multidisciplinary approach in which specialists in diagnosis, surgery, chemotherapy, and radiation therapy must work collaboratively [10-12]. Management of advanced cancer requires a high level of expertise and coordination, which can only be achieved through years of rigorous training and clinical experience. However, the ongoing healthcare crisis in South Korea has persisted for over 1 year, significantly disrupting essential training pipelines. If this situation remains unresolved, the long-term capacity to deliver high-quality cancer care will be jeopardized. Immediate action is required to resolve this crisis before its impact becomes irreversible. Ensuring a sustainable healthcare workforce is not just about resolving current disruptions but also about safeguarding the future of patient care in South Korea.

This study had several limitations. First, although its retrospective design allows for an analysis of real-world changes in clinical practice, it does not establish definitive conclusions regarding the causality between policy changes and observed outcomes. Second, the study was conducted at two national university hospitals, which may limit the generalizability of the findings to other healthcare settings. The capacity to manage the medical crisis may have differed depending on the type of hospital (national university hospital, private university hospital, or private hospital), as well as the hospital’s role within its regional healthcare system. Third, although this study identified differences in treatment timing and short-term clinical outcomes, long-term oncological outcomes, including overall survival and disease-free survival, were not evaluated. Future research should investigate the long-term consequences of delayed colorectal cancer treatment and assess potential mitigation strategies to ensure continuity of cancer care during disruptions in the healthcare system.

In conclusion, this study demonstrated that the abrupt policy to increase medical school admissions and subsequent workforce crisis significantly influenced the timing of colorectal cancer surgeries, initial treatment decisions, and postoperative outcomes in two tertiary hospitals in South Korea. These findings emphasize the need for carefully planned healthcare policies that prioritize workforce stability and ensure uninterrupted access to essential cancer care services.

## Figures and Tables

**Table 1.** Initial treatment modalities and time to treatment initiation in the entire cohort

Characteristic	March to August 2023	March to August 2024	*p*-value
No. of patients	895	853	
Age (yr)	66.0 (58.0–75.0)	65.0 (58.0–75.0)	0.568
Sex, male/female	568/327	518/335	0.259
ASA grade			0.001
I	31 (3.9)	54 (11.8)	
II	515 (64.3)	317 (69.4)	
III	250 (31.2)	86 (18.8)	
IV	2 (0.2)	0 (0)	
Unknown	3 (0.4)	0 (0)	
Colon/rectum/synchronous	506/377/12	541/302/10	0.013
CEA (ng/mL)	2.8 (1.8–5.8)	2.4 (1.4–6.3)	0.001
Clinical T stage			0.015
T0–2	228 (25.5)	176 (20.6)	
T3, T4	555 (62.0)	540 (63.3)	
Unknown	112 (12.5)	137 (16.1)	
Clinical N stage			0.001
N0	432 (48.3)	318 (37.3)	
N1, N2	341 (38.1)	398 (46.7)	
Unknown	122 (13.6)	137 (16.1)	
Clinical M1	43 (4.8)	68 (8.0)	0.002
Treatment			0.001
Upfront surgery	569 (63.5)	337 (39.5)	
Chemotherapy	111 (12.4)	76 (8.9)	
Chemoradiotherapy	155 (17.3)	157 (18.4)	
Others^a)^	60 (6.7)	283 (33.2)	
Observation	9 (15.0)	12 (4.2)	
Endoscopic resection	35 (58.3)	33 (11.7)	
Referral to another hospital	16 (26.7)	238 (84.1)	
Treatment, cT0–2			0.001
Upfront surgery	209 (93.3)	96 (80.0)	
Chemotherapy	4 (1.8)	4 (3.3)	
Chemoradiotherapy	11 (4.9)	20 (16.7)	
Treatment, cT3–4/others			0.001
Upfront surgery	360 (58.9)	241 (53.6)	
Chemotherapy	107 (17.5)	72 (16.0)	
Chemoradiotherapy	144 (23.6)	137 (30.4)	
Duration (day)^b)^	29.0 (19.0–48.0)	22.0 (3.0–52.0)	0.001
Duration in the same hospital (day)^c)^	30.0 (21.0–50.0)	36.0 (22.0–68.0)	0.001

Values are presented as number only, median (interquartile range), or number (%). ASA, American Society of Anesthesiologists physical status classification; CEA, carcinoembryonic antigen.

a) Patient underwent observation, received endoscopic resection, or was referred to another hospital.

b) Period from the first outpatient visit to the start of treatment or referral to another hospital.

c) Period from the first outpatient visit to the start of treatment.

**Table 2.** Clinical and pathological stage and time to surgery among patients who underwent upfront resection

Characteristc	March to August 2023	March to August 2024	*p*-value
No. of patients	569	337	
Age (yr)	67.0 (59.0–76.0)	66.0 (60.0–75.0)	0.678
Sex, male/female	356/213	207/130	0.786
ASA grade			0.001
I	15 (2.6)	26 (7.7)	
II	360 (63.3)	241 (71.5)	
III	189 (33.2)	70 (20.8)	
IV	2 (0.4)	0 (0)	
Unknown	3 (0.5)	0 (0)	
Colon/rectum/synchronous	409/148/12	270/61/6	0.021
CEA (ng/mL)	2.7 (1.8–4.9)	2.1 (1.3–4.3)	<0.001
Clinical T stage			0.018
T0–2	209 (36.7)	96 (28.5)	
T3, T4	284 (49.9)	200 (59.3)	
Unknown	76 (13.4)	41 (12.2)	
Clinical N stage			<0.001
N0	346 (60.8)	123 (36.5)	
N+	147 (25.9)	173 (51.3)	
Unknown	76 (13.4)	41 (12.2)	
Clinical M1	6 (1.1)	8 (2.4)	0.045
Duration (day)^a)^	30.0 (22.0–44.0)	52.0 (30.0–72.0)	<0.001
Hospital stays (day)	7.0 (6.0–8.0)	7.0 (6.0–9.0)	0.001
Operative approach			0.018
Laparoscopic	436 (76.6)	235 (69.7)	
Robotic	96 (16.9)	83 (24.6)	
Open	37 (6.5)	19 (5.6)	
Operative time (min)	137.0 (80.0– 103.0)	120.0 (95.0–160.0)	<0.001
Estimated blood loss (mL)	10.0 (10.0–30.0)	10.0 (10.0–30.0)	0.797
Postoperative complications	68 (12.0)	95 (28.2)	<0.001
CD grade I	3 (4.4)	14 (14.7)	
CD grade II	47 (69.1)	65 (68.4)	
CD grade III	18 (26.5)	14 (14.7)	
CD grade IV	0 (0)	2 (2.1)	
Pathologic stage			0.002
0	19 (3.3)	8 (2.4)	
I	160 (28.1)	61 (18.1)	
II	173 (30.4)	101 (30.0)	
III	201 (35.3)	158 (46.9)	
IV	16 (2.8)	9 (2.7)	
Differentiation			0.075
Well	74 (13.0)	42 (12.5)	
Moderate	418 (73.5)	270 (80.1)	
Poor	36 (6.3)	12 (3.6)	
Mucinous/signet-ring	7 (1.2)	3 (0.9)	
Unknown	34 (6.0)	10 (3.0)	
Lymphovascular invasion	161 (28.3)	100 (29.7)	0.065
Venous invasion	219 (38.5)	139 (41.2)	0.055
Perineural invasion	292 (51.3)	209 (62.0)	0.001

Values are presented as number only, median (interquartile range), or number (%). ASA, American Society of Anesthesiologists physical status classification; CEA, carcinoembryonic antigen; CD, Clavien–Dindo classification.

a) Period from the first outpatient visit to the start of treatment.

**Table 3.** Univariate and multivariable analysis of risk factors for postoperative complications (2020–2024)

Risk factor	Univariate		Multivariable	
	HR (95% CI)	*p*-value	HR (95% CI)	*p*-value
Male sex (ref, female)	2.03 (1.56–2.64)	<0.001	1.96 1.51–2.57)	<0.001
Age <60 yr	0.96 (0.74–1.25)	0.800		
ASA grade III, IV	0.87 (0.66–1.13)	0.300		
Location, rectum (ref, colon)	1.65 (1.27–2.12)	<0.001	1.80 (1.38–2.34)	<0.001
Year of surgery, 2024 (ref, 2020–2023)	3.24 (2.46–4.26)	<0.001	3.50 (2.64–4.64)	<0.001
cT3, cT4	0.84 (0.57–1.23)	0.400		
cN1, cN2	1.07 (0.83–1.39)	0.600		
Distant metastasis	1.00 (1.00–1.00)	0.800		

HR, hazard ratio; CI, confidence interval; ASA, American Society of Anesthesiologists physical status classification.

## Supplementary Materials

Supplementary Tables 1 and 2 can be found at https://doi.org/10.12701/jyms.2026.43.4.

Supplementary Table 1. Initial treatment modalities and treatment duration in the entire cohort

Supplementary Table 2. Clinical and pathological stage and time to surgery in patients who underwent upfront surgery
