# Supplementary material for: Health system disruption and oncologic consequences: a retrospective observational study of South Korea’s 2024 medical walkout
Source: J Yeungnam Med Sci. 2025 Dec 18;43:4. doi: 10.12701/jyms.2026.43.4 (PMC12887129; doi:10.12701/jyms.2026.43.4)
Supplement: Supplementary Table 1. — Initial treatment modalities and treatment duration in the entire cohort [file jyms-2026-43-4-Supplementary-Table-1.pdf]

Supplementary Table 1. Initial treatment modalities and treatment duration in the entire cohort

Characteristic	March to August 2020 (n = 682)	March to August 2021 (n = 833)	March to August 2022 (n = 859)	March to August 2023 (n = 895)	March to August 2024 (n = 853)	*p*-value
Age (yr)	68.0 (57.0–76.0)	67.0 (59.0–76.0)	66.0 (58.0–75.0)	66.0 (58.0–75.0)	65.0 (58.0–75.0)	0.729
Sex, male/female	412/270	488/345	529/330	568/327	518/335	0.333
ASA grade						< 0.001
I	77 (12.7)	49 (6.5)	61 (7.8)	31 (3.9)	54 (11.8)	
II	399 (65.6)	501 (66.4)	512 (65.7)	515 (64.3)	317 (69.4)	
III	125 (20.6)	196 (26.0)	195 (25.0)	250 (31.2)	86 (18.8)	
IV	3 (0.5)	8 (1.1)	11 (1.4)	2 (0.2)	0 (0)	
Unknown	4 (0.7)	1 (0.1)	0 (0)	3 (0.4)	0 (0)	
Colon/rectum/synchronous	393/285/4	484/324/25	469/377/13	506/377/12	541/302/10	< 0.001
CEA (ng/mL)	2.8 (1.7–5.8)	2.6 (1.5–6.1)	3.0 (1.6–7.7)	2.8 (1.8–5.8)	2.4 (1.4–6.3)	0.016
Clinical T stage						< 0.001
T0–2	123 (18.0)	160 (19.2)	146 (17.0)	228 (25.5)	176 (20.6)	
T3, T4	485 (71.1)	543 (65.2)	589 (68.6)	555 (62.0)	540 (63.3)	
Unknown	74 (10.9)	130 (15.6)	124 (14.4)	112 (12.5)	137 (16.1)	
Clinical N stage						< 0.001
N0	264 (38.7)	309 (37.1)	348 (40.5)	432 (48.3)	318 (37.3)	
N1, N2	344 (50.4)	394 (47.3)	383 (44.6)	341 (38.1)	398 (46.7)	
Unknown	74 (10.9)	130 (15.6)	128 (14.9)	122 (13.6)	137 (16.1)	
Clinical M1	57 (8.4)	65 (7.8)	97 (11.3)	43 (4.8)	68 (8.0)	< 0.001
Treatment						< 0.001
Upfront surgery	468 (68.6)	525 (63.0)	493 (57.4)	569 (63.5)	337 (39.5)	
Chemotherapy	72 (10.6)	120 (14.4)	154 (17.9)	111 (12.4)	76 (8.9)	
Chemoradiotherapy	98 (14.4)	128 (15.4)	142 (16.5)	155 (17.3)	157 (18.4)	
Others[a]	44 (6.5)	60 (7.2)	70 (8.1)	60 (6.7)	283 (33.2)	
Treatment, cT0–2						< 0.001
Upfront surgery	91 (91.9)	136 (90.7)	132 (93.0)	209 (93.3)	96 (80.0)	
Chemotherapy	0 (0)	0 (0)	6 (4.2)	4 (1.8)	4 (3.3)	
Chemoradiotherapy	8 (8.1)	14 (9.3)	4 (2.8)	11 (4.9)	20 (16.7)	
Treatment, cT3–4/others						< 0.001
Upfront surgery	377 (69.9)	389 (62.4)	361 (55.8)	360 (58.9)	241 (53.6)	
Chemotherapy	72 (13.4)	120 (19.3)	148 (22.9)	107 (17.5)	72 (16.0)	
Chemoradiotherapy	90 (16.7)	114 (18.3)	138 (21.3)	144 (23.6)	137 (30.4)	
Duration (day)[b]	18.5 (9.0–29.0)	22.0 (12.0–36.0)	27.0 (15.0–57.0)	29.0 (19.0–48.0)	22.0 (3.0–52.0)	< 0.001
Duration, the same hospital (day)[c]	19.5 (11.0–30.0)	23.0 (14.0–39.0)	29.0 (18.0–75.0)	30.0 (21.0–50.0)	36.0 (22.0–68.0)	< 0.001

Values are presented as median (interquartile range) or number (%).
ASA, American Society of Anesthesiologists physical status classification; CEA, carcinoembryonic antigen
[a]Patient underwent observation, received endoscopic resection, or was referred to another hospital. [b]Period from the first outpatient visit to the start of treatment or referral to another hospital.
[c]Period from the first outpatient visit to the start of treatment.
