# Supplementary material for: Health system disruption and oncologic consequences: a retrospective observational study of South Korea’s 2024 medical walkout
Source: J Yeungnam Med Sci. 2025 Dec 18;43:4. doi: 10.12701/jyms.2026.43.4 (PMC12887129; doi:10.12701/jyms.2026.43.4)
Supplement: Supplementary Table 2. — Clinical and pathological stage and time to surgery in patients who underwent upfront surgery [file jyms-2026-43-4-Supplementary-Table-2.pdf]

Supplementary Table 2. Clinical and pathological stage and time to surgery in patients who underwent upfront surgery

Characteristic	March to August 2020 (n = 468)	March to August 2021 (n = 525)	March to August 2022 (n = 493)	March to August 2023 (n = 569)	March to August 2024 (n = 337)	*p*-value
Age (yr)	71.0 (59.0–77.0)	69.0 (60.0–77.0)	69.0 (60.0–77.0)	67.0 (59.0–76.0)	66.0 (60.0–75.0)	0.607
Sex, male/female	272/196	290/235	289/204	356/213	207/130	0.133
ASA grade						< 0.001
I	41 (8.8)	25 (4.8)	35 (7.1)	15 (2.6)	26 (7.7)	
II	305 (65.2)	343 (65.3)	308 (62.5 5)	360 (63.3)	241 (71.5)	
III	115 (24.6)	150 (28.6)	143 (29.0)	189 (33.2)	70 (20.8)	
IV	3 (0.6)	6 (1.1)	7 (1.4)	2 (0.4)	0 (0)	
Unknown	4 (0.9)	1 (0.2)	0 (0)	3 (0.5)	0 (0)	
Colon/rectum/synchronous	319/147/2	366/142/17	349/135/9	409/148/12	270/61/6	< 0.001
CEA (ng/mL)	2.7 (1.7–5.0)	2.4 (1.5–4.7)	2.6 (1.5–5.3)	2.7 (1.8–4.9)	2.1 (1.3–4.3)	0.007
Clinical T stage						< 0.001
T0–2	91 (19.4)	136 (25.9)	132 (26.8)	209 (36.7)	96 (28.5)	
T3, T4	318 (67.9)	289 (55.1)	269 (54.6)	284 (49.9)	200 (59.3)	
Unknown	59 (12.6)	100 (19.0)	92 (18.7)	76 (13.4)	41 (12.2)	
Clinical N stage						< 0.001
N0	216 (46.2)	245 (46.7)	264 (53.5)	346 (60.8)	123 (36.5)	
N1, N2	194 (41.5)	180 (34.3)	137 (27.8)	147 (25.9)	173 (51.3)	
Unknown	58 (12.4)	100 (19.0)	92 (18.7)	76 (13.4)	41 (12.2)	
Clinical M1	11 (2.4)	15 (2.9)	11 (2.2)	6 (1.1)	8 (2.4)	0.002
Duration (day)[a)]	22.0 (14.0–36.0)	23.0 (16.0–36.0)	29.0 (19.0–41.0)	30.0 (22.0–44.0)	52.0 (30.0–72.0)	< 0.001
Hospital stays (day)	7.0 (6.0–8.0)	7.0 (6.0–8.0)	7.0 (6.0–8.0)	7.0 (6.0–8.0)	7.0 (6.0–9.0)	< 0.001
Postoperative complications	82 (17.5)	36 (6.9)	36 (7.3)	68 (12.0)	95 (28.2)	< 0.001
CD grade I	9 (11.0)	10 (27.8)	6 (16.7)	3 (4.4)	14 (14.7)	
CD grade II	60 (73.2)	10 (27.8)	16 (44.4)	47 (69.1)	65 (68.4)	
CD grade III	12 (14.6)	14 (38.9)	12 (33.3)	18 (26.5)	14 (14.7)	
CD grade IV	1 (1.2)	2 (5.6)	2 (5.6)	0 (0)	2 (2.1)	
Pathologic stage						< 0.001
0	8 (1.7)	27 (5.1)	18 (3.7)	19 (3.3)	8 (2.4)	
1	108 (23.1)	138 (26.3)	131 (26.6)	160 (28.1)	61 (18.1)	
2	171 (36.5)	164 (31.2)	143 (29.0)	173 (30.4)	101 (30.0)	
3	151 (32.3)	160 (30.5)	170 (34.5)	201 (35.3)	158 (46.9)	
4	30 (6.4)	36 (6.9)	31 (6.3)	16 (2.8)	9 (2.7)	
Differentiation						< 0.001
Well	105 (22.4)	102 (19.4)	82 (16.6)	74 (13.0)	42 (12.5)	
Moderate	325 (69.4)	358 (68.2)	327 (66.3)	418 (73.5)	270 (80.1)	
Poor	34 (7.3)	51 (9.7)	35 (7.1)	36 (6.3)	12 (3.6)	
Mucinous or SRC	4 (0.9)	4 (0.8)	6 (1.2)	7 (1.2)	3 (0.9)	
Unknown	0 (0)	10 (1.9)	43 (8.7)	34 (6.0)	10 (3.0)	
Lymphovascular invasion	85 (18.2)	116 (22.1)	126 (25.6)	161 (28.3)	100 (29.7)	< 0.001

(Continued to the next page)

Supplementary Table 2. (Continued)

Characteristic	March to August 2020 (n = 468)	March to August 2021 (n = 525)	March to August 2022 (n = 493)	March to August 2023 (n = 569)	March to August 2024 (n = 337)	*p*-value
Venous invasion	126 (26.9)	184 (35.0)	193 (39.1)	219 (38.5)	139 (41.2)	< 0.001
Perineural invasion	233 (49.8)	259 (49.3)	244 (49.5)	292 (51.3)	209 (62.0)	< 0.001

Values are presented as median (interquartile range) or number (%).
ASA, American Society of Anesthesiologists physical status classification; CEA, carcinoembryonic antigen; CD, Clavien–Dindo classification; SRC, signet ring cell.
[a]Period from the first outpatient clinic visit to the start of treatment.
